# The procedural learning deficit hypothesis of language learning disorders: we see some problems

**DOI:** 10.1111/desc.12552

**Published:** 2017-03-02

**Authors:** Gillian West, Miguel A. Vadillo, David R. Shanks, Charles Hulme

**Affiliations:** ^1^ Department of Language and Cognition University College London UK; ^2^ Department of Primary Care and Public Health Sciences King's College London UK; ^3^ Department of Experimental Psychology University College London UK

## Abstract

Impaired procedural learning has been suggested as a possible cause of developmental dyslexia (DD) and specific language impairment (SLI). This study examined the relationship between measures of verbal and non‐verbal implicit and explicit learning and measures of language, literacy and arithmetic attainment in a large sample of 7 to 8‐year‐old children. Measures of verbal explicit learning were correlated with measures of attainment. In contrast, no relationships between measures of implicit learning and attainment were found. Critically, the reliability of the implicit learning tasks was poor. Our results show that measures of procedural learning, as currently used, are typically unreliable and insensitive to individual differences. A video abstract of this article can be viewed at: https://www.youtube.com/watch?v=YnvV-BvNWSo

## RESEARCH HIGHLIGHTS

- This study examined the relationships between procedural and declarative memory skills and language attainment in a large, unselected sample of 7 to 8‐year‐old children.
- Verbal declarative memory measures correlated with language attainment.
- Crucially, the procedural memory measures demonstrated very low reliability and did not correlate with each other or with measures of attainment.
- These results raise concerns about claims that a procedural deficit can be adequately assessed, and cast doubts on claims that there may be a causal relationship between procedural learning deficits and language learning disorders.

## 1 INTRODUCTION

According to the procedural deficit hypothesis (Nicolson & Fawcett, 2007, 2011; Ullman, 2004; Ullman & Pierpont, 2005), a key risk factor for language learning disorders such as developmental dyslexia (DD) and specific language impairment (SLI) is impaired procedural learning. However, as we will document below, studies evaluating this hypothesis have produced highly inconsistent results. We believe such inconsistencies may reflect a reliance on measures with low reliability and the use of extreme group designs with small group sizes. In the current paper we take a different approach to this issue: we assess the relationships between measures of language and attainment and a wide range of measures of both procedural and declarative learning in a large unselected sample of children. We also take care to assess the reliabilities of all measures used.

The procedural deficit hypothesis takes a dual process view of memory as its starting point (Squire, 2004). According to this view, the declarative memory system, which is involved in the acquisition, storage and use of facts and events, is the foundation for the creation of a mental lexicon which stores word‐specific knowledge (Ullman, 2004). In contrast, the procedural memory system regulates the acquisition, consolidation and automization of motor, perceptual and cognitive skills (Lum, Gelgic, & Conti‐Ramsden, 2010). In language, it underpins the learning of a ‘mental Grammar’, which is concerned with the rule‐based procedures that govern the regularities of language (Chomsky, 1980; Ullman, 2004). The procedural deficit hypothesis suggests that it is a deficit in procedural sequence learning that is a critical cognitive risk factor for dyslexia and language impairment (Nicholson & Fawcett, 2010), while declarative learning mechanisms remain relatively intact. The theory suggests that problems in a procedural learning system should be found in different modalities (Ullman, 2004), affecting both non‐verbal and verbal stimuli.

It may be useful to briefly consider terminology. The terms procedural and implicit are largely synonymous (Shanks, 2005; Berry & Dienes, 1993), but a concise definition of the distinction between implicit and explicit learning is not straightforward (Frensch & Runger, 2003). Reber, Walkenfeld, and Hernstadt's (1991) definition states that in implicit learning both learning and the resulting knowledge are dissociated from awareness. Explicit learning on the other hand uses deliberate strategies, is accessible to consciousness and can be reported upon demand (Shanks, 2005). In what follows, implicit learning and procedural learning will be used interchangeably, as will explicit and declarative learning.

Research on the relationship between language skills and explicit memory skills has frequently used free recall and serial recall tasks. Impaired free recall (Menghini, Carlesimo, Marotta, Finzi, & Vicari, 2010, Vellutino & Scanlon, 1985) and serial recall (Di Betta & Romani, 2006; Perez, Majerus, Mahot, & Poncelet, 2012) have been found in adults and children with language‐learning disorders.

Research on the relationship between language skills and implicit learning has used a variety of tasks ranging from artificial grammar learning (Reber, 1967) to mirror‐drawing (Vicari et al., 2005). Support for the procedural deficit view comes mainly from extreme group designs showing impaired performance of language‐disordered participants on implicit serial learning tasks. The most widely used such measure is the non‐verbal serial reaction time task (SRT; Nissen & Bullemer, 1987). In this task participants respond as quickly as possible to a visual stimulus appearing in one of four locations on a screen. Faster responding to trials that follow a covert sequence compared to random trials is taken as evidence of implicit learning (Seger, 1994). The original deterministically structured serial reaction time task has been criticized for not fully dissociating implicit and explicit learning (Shanks & Johnstone, 1999). More complex, probabilistically structured (Schvaneveldt & Gomez, 1998) or alternating versions (Howard & Howard, 1997) have been developed to minimize the risk of explicit learning. Language‐disordered children have been reported to perform poorly both on deterministic serial reaction time tasks (Jiménez‐Fernández, Vaquero, Jiménez, & Defior, 2011; Lum et al., 2010; Lum, Ullman, & Conti‐Ramsden, 2013; Vicari et al., 2005) and more complex alternating versions of the task (Hedenius, 2013; Howard, Howard, Japikse, & Eden, 2006). However, findings are mixed with null results in some studies of adults with dyslexia (Kelly, Griffiths, & Frith, 2002; Rüsseler, Gerth, & Münthe, 2006), and children with SLI (Gabriel, Maillart, Guillaume, Stefaniak, & Meulemans, 2011; Lum & Bleses, 2012).

The contextual cueing task (Chun & Jiang, 1998) is another non‐verbal measure of implicit learning (Goujon, Didierjean, & Thorpe, 2015). In this task, participants are instructed to find the location of a target stimulus within matrices of distractor stimuli. The position of the target in some matrices is predictable, and faster responding to these compared to random unpredictable matrices is considered evidence of implicit learning. Implicit learning in contextual cueing has been found in typically developing children (Dixon, Zelazo, & De Rosa, 2010; Merrill, Conners, Roskos, Klinger, & Klinger, 2013), although the degree to which it is present in childhood is disputed (Couperus, Hunt, Nelson, & Thomas, 2011). However, studies have so far not found impaired performance in dyslexic adults (Bennett, Romano, Howard, & Howard, 2008; Howard et al., 2006) or children (Jiménez‐Fernández et al., 2011), although impaired implicit sequence learning was found in these same participants.

The most widely used measure of verbal implicit learning is the Hebb serial order learning task (Hebb, 1961). In this task participants perform a verbal serial recall task, where they are asked to recall lists of words in the order of presentation; unknown to the participants, a repeating sequence is introduced. Better recall of the repeated, compared to non‐repeated, sequences provides evidence of implicit learning. Poor implicit learning on this task has been found in children with SLI (Hsu & Bishop, 2014) and in dyslexic adults (Bogaerts, Szmalec, Hachmann, Page, & Duyck, 2015; Szmalec, Loncke, Page, & Duyck, 2011). Szmalec et al. (2011) also found dyslexic adults to be impaired on a non‐verbal visuo‐spatial Hebb task using sequences of dot locations, suggestive of a domain‐general impairment. However, once again findings are mixed and Staels and Van den Broeck (2015) found no evidence of impaired learning on a verbal Hebb task in adolescents or children with dyslexia and nor did Majerus et al. (2009) in a study of children with SLI.

There are a number of possible reasons for the inconsistent results from studies of the relationship between implicit learning and language learning disorders. The vast majority of studies use extreme group designs. Yet, dyslexia and specific language impairment are dimensional, heterogenous, often co‐morbid, neuro‐developmental disorders (Bishop & Snowling, 2004; Peterson & Pennington, 2015). Language‐disordered groups from different studies may not, therefore, reflect the same behavioural symptoms or underlying cognitive impairments. Extreme group designs also tend to overestimate the size of any linear association between variables (Preacher, 2015; Preacher, Rucker, MacCallum, & Nicewander, 2005) and potentially produce measures that may be lower in reliability (Preacher, 2015). In addition, given the difficulties inherent in recruitment and testing of language‐disordered participants, sample sizes in these studies are typically small, further reducing confidence in results. Finally, there are reasons to suspect that the implicit memory tasks themselves may not be reliable (Buchner & Wippich, 2000; Reber et al., 1991; Salthouse, McGuthry, & Hambrick, 1999) and tasks with poor reliability produce large errors of measurement and are inherently insensitive to individual differences (Nunnally & Bernstein, 1994). However, previous studies have rarely, if ever, reported the reliability of the tasks used to measure implicit learning.

In summary, it has been suggested that language learning impairments (specific language impairment and dyslexia) may reflect a procedural learning deficit. A variety of different tasks, involving both verbal and non‐verbal stimuli, have been used to assess implicit learning in groups with language learning impairments with inconsistent results. An important question is whether the different measures of implicit learning used to investigate procedural learning really do measure a common underlying procedural learning system, which is distinct from a declarative memory system. Another important question is whether the tasks currently used to assess implicit learning are reliable.

The current study uses a large sample of children unselected for ability. This has the advantage that it will not over‐estimate the size of any association between measures of attainment and memory performance, as an extreme groups design might. It also uses multiple measures of implicit memory (the serial reaction time, Hebb serial learning and contextual cueing tasks) and explicit memory (immediate serial recall and free recall tasks), using both verbal and non‐verbal stimuli. Using this wide range of tasks in a concurrent correlational design will allow us to assess the factor structure of the tasks and explore whether there are separable implicit and explicit memory systems. We will then be able to assess the extent to which variations in language and reading skills are correlated with variations in implicit or explicit memory skills, should these be dissociable. We will also determine the reliability of the different measures which is imperative when investigating individual differences.

## 2 METHOD

### 2.1 Design

This is a concurrent correlational study investigating the possible associations between language attainment and explicit and implicit memory skills in 7‐ and 8‐year‐old children.

### 2.2 Participants

Ethical clearance for the study was provided by the UCL Research Ethics committee. One hundred and one Year 3 children (64 girls, 37 boys) from three London primary schools took part. Children's ages ranged from 7 years 5 months to 8 years 7 months (mean = 8 years and 1 month; *SD* = 3.82 months). Fifty‐two of the participating children used English as an additional language but were judged by their class teachers to be fluent in English.

### 2.3 Tasks and testing procedures

All children completed a battery of attainment measures that was administered in a single session to whole classes. Subsequently, children completed three further individual testing sessions. The final session comprised four tasks the children had completed before (verbal and non‐verbal versions of declarative and implicit memory tasks) in order to measure memory consolidation. Tasks were administered in a fixed order to all children.

#### 2.3.1 Attainment tasks

##### Test of receptive grammar (TROG‐2; Bishop, 2003)

This was adapted for group administration. Children were asked to match spoken sentences to one of four pictures.

##### Wide Range Achievement spelling subtest (WRAT‐3; Wilkinson, 1993)

Children were asked to spell 15 words (go, cat, boy, run, will, cut, arm, dress, train, shout, watch, grown, kitchen result, heaven) that were dictated by the experimenter.

##### Picture Word Matching (PWM; Caravolas et al., 2012)

This timed single word reading test consisted of 63 items, each of which showed a picture of an object or scene with four printed words (the correct word and three distractor words). Children were given 3 minutes to select the correct word for as many items as possible.

##### Test of word and non‐word reading efficiency (TOWRE‐2; Torgesen, Wagner, & Rashotte, 1999)

These individually administered tests required children to read aloud as many words (or non‐words) as they could in 45 seconds.

##### Test of basic arithmetic and number skills (TOBANS; Brigstocke, Moll, & Hulme, 2016)

These timed tests were designed to assess fluency in addition, subtraction, and multiplication, giving a composite arithmetic score. In addition, dot and digit comparison tasks required children to circle the larger of two groups of dots or the larger of two Arabic numerals, respectively. Finally, a test assessed the speed and accuracy of counting random arrays of dots. The TOBANS subtests had no reading requirement, with all instructions read aloud to the children.

##### WASI (Wechsler, 1999)

The WASI matrix reasoning subtest (Wechsler, 1999) was used to assess non‐verbal ability.

#### 2.3.2 Declarative memory tasks

##### Word lists (Cohen, 1997)

This free recall test from the Children's Memory Scale assessed children's ability to learn a list of 10 unrelated words over four learning trials. Children were asked to recall as many words as possible in any order from a list of 10 unrelated words read out by the experimenter (Trial 1). After the first trial only words that had been omitted were read out to children for each of the following three trials (Trials 2–4). Children were then asked to recall a distractor list of 10 different words that were spoken by the examiner. A final trial on the first list (without re‐presentation of the list) was then attempted (Trial 5). The score for the first five trials formed the child's Learning Score. A measure of delayed recall was taken by asking the child to recall the list once more at the end of the testing session (Trial 6). A final memory consolidation measure was taken during the last testing session several days later, asking children to recall as many words as possible from the 10‐item list (Trial 7). Scheduling constraints meant the time lapse between Trial 6 and 7 was not the same for all children, but restricting inclusion to the majority of participants with a two‐day lapse did not significantly alter results.

##### Dot Locations (Cohen, 1997)

The Dot Locations task from the Children's Memory Scale was used as a non‐verbal analogue of the Word Lists free recall task. It tested recall of a static dot pattern configuration, giving a measure of declarative, non‐verbal spatial memory. Children were shown a 4 × 3 grid with a pattern of six red dots for 5 seconds. Children were then asked to re‐create it on an empty grid, using red plastic discs (Trial 1). This was repeated twice (Trials 2–3). A distractor pattern of yellow dots was then shown and the children were asked to reproduce it. Without re‐presenting the first pattern, children were then asked to reproduce it once again (Trial 4). A point was scored for each correct location on each attempt. The mean of the scores for these four trials formed the child's learning score. Delayed recall was tested by asking the children to reproduce the initial configuration at the end of the testing session (Trial 5). A memory consolidation measure was taken during the final session (Trial 6), asking the children to reproduce the pattern once more. Again, the time lapse between Trials 5 and 6 was not the same for all children, but all were included in analysis, as restricting inclusion did not significantly alter results.

##### Immediate serial recall (ISR)

These tasks were developed to give declarative verbal and non‐verbal measures that specifically targeted memory for sequences. They formed the beginning of the implicit memory Hebb sequence learning tasks.

**Figure 1:**
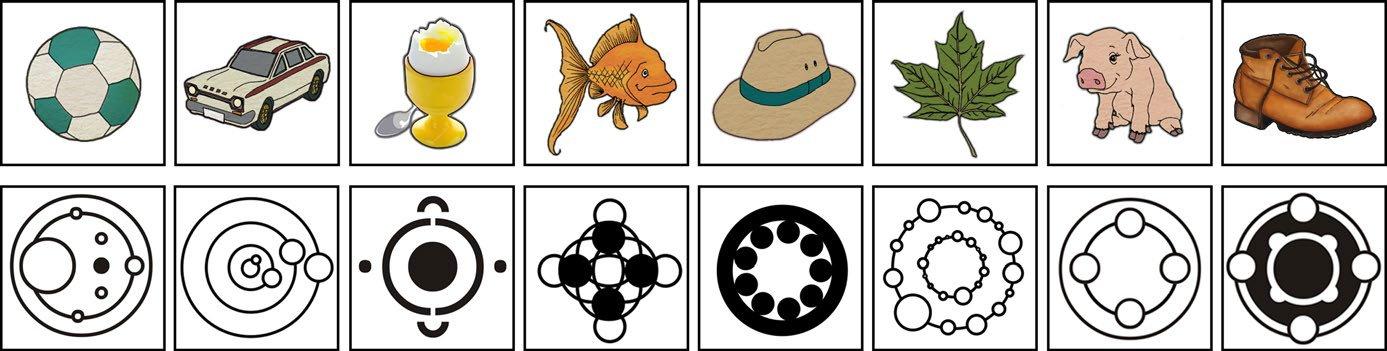
Immediate serial recall and Hebb task verbal and non‐verbal stimuli

Two versions of the task were created: a verbal task that used nameable pictures as stimuli and a non‐verbal task that used abstract symbols. A total of eight stimuli were used for each version of the task. The non‐verbal and verbal stimuli used are shown in Figure 1.

Eight pictures with dissimilar names were selected that 7–8‐year‐old children would be familiar with (fish, car, egg, shoe, pig, hat, leaf, ball). Symbols for the non‐verbal condition were selected that were judged to be difficult to name but were easily discriminable from each other (http://www.dudeman.net/siriusly/cc/phenom.html). Verbal and non‐verbal versions were administered as separate tasks during different testing sessions.

On each trial a sequence of stimuli was presented across the top of a computer screen. All eight possible stimuli then appeared across the middle of the screen in a random order. Children were instructed to use the computer mouse to click on these stimuli to reconstruct the sequence they had just seen. Each item the child clicked on disappeared from the central display, reappearing in the order of selection in the child's reconstructed list at the bottom of the screen. Once an item was selected it could not be changed. All trial sequences were randomly generated.

The task began with an eight‐trial practice round with each trial presenting a single stimulus. The recorded portion of the task began with four trials at sequence length 2. If the child reconstructed one or more of these sequences correctly they proceeded to the next level (three‐item sequences). Each subsequent level contained four trials, at a sequence length one item longer than the preceding level up to a maximum of seven items. Trials continued until all four trials at a given sequence length were incorrectly reconstructed, at which point testing stopped. At each increase in sequence length the test sequence remained on the screen for an additional 1 second, starting at 3 seconds for two‐item sequences. The number of trials correctly reconstructed at each sequence length was recorded. This information was used to calculate a span score, consisting of the longest sequence length recalled correctly on all four trials, plus .25 for each longer sequence correctly recalled (see Conway et al., 2005; Hulme, Maughan, & Brown, 1991).

#### 2.3.3 Implicit memory tasks

All implicit memory tasks were presented on a Dell laptop with a 15 inch screen with resolution set at 1366 × 768 dpi.

##### Serial Reaction Time task (SRT)

An SRT task (Nissen & Bullemer, 1987) with a probabilistic sequence structure based on Schvaneveldt and Gomez (1998) was used to investigate non‐verbal implicit spatial sequence learning. A verbal analogue of the SRT task adapted from Hartman, Knopman, and Nissen (1989) was devised to test verbal implicit sequence learning.

For the non‐verbal SRT task (NV‐SRT) two 12‐item sequences were taken from Shanks, Wilkinson, and Channon (2003): sequence A – 314324213412; sequence B – 431241321423. In both sequences, each location repeated three times, each time being preceded by a different location; each sequence contained one reversal (121 or 343) and no repeated locations. They differed only in their second‐order conditional structure. Each block started with a randomly chosen bigram, e.g., 3 2. The next location selected was either the location that followed that bigram in sequence A (with a probability of .9, i.e., 4), or was the location that followed the bigram in Sequence B (with a probability of .1, i.e., 1). This process then repeated with the new most recent bigram, either 2 4, if the transition had been a probable one, or 2 1 if the transition had been improbable. The task continued in this way until the end of the block.

**Figure 2:**
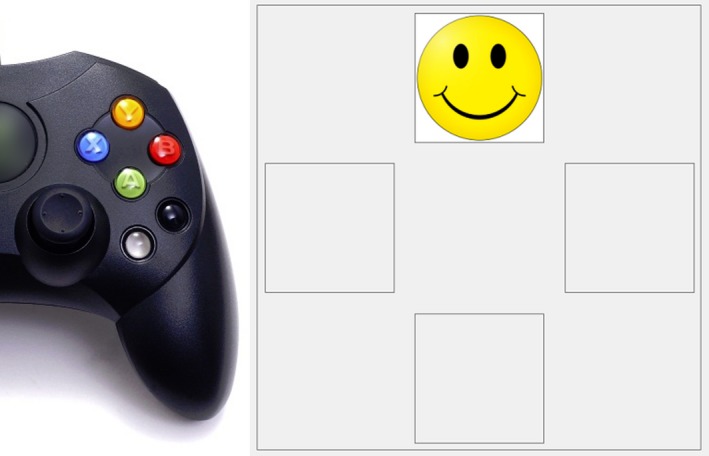
Non‐verbal serial reaction time task. Children pressed the button on the controller that matched the location of the stimulus

Children were seated in front of a laptop connected to an Xbox Gamepad controller. For each trial a stimulus of a smiley yellow face appeared on the screen in one of four locations. The locations formed a diamond pattern that corresponded to the pattern of buttons on the Gamepad controller (see Figure 2). The children were told to press the button that corresponded to the position of each stimulus as quickly as possible. There were 500 trials. Ten practice trials began, with equal probabilities of each sequence occurring. There were then five blocks of 100 trials that followed the sequence probabilities outlined above.

The program recorded the RT and the button pressed, whether correct or incorrect, but required the child to press the correct button before going on to the next trial. There was a 250‐ms interval between trials. A pause between blocks allowed the child a short break if needed, with the experimenter manually starting each new block as soon as the child was ready to continue. The task took approximately 15 minutes to complete. Faster RTs for probable compared to improbable transitions were taken as evidence of implicit learning.

For the verbal SRT task (V‐SRT) the sequences were the same as those used by Schwaneveldt and Gomez (1998; Probable sequence A: 121342314324; Improbable sequence B: 123413214243). The probabilistic structure of the task was otherwise identical to the NV‐SRT. This task used four nameable pictures as stimuli (bird, hammer, fish, tree). The pictures were approximately 10 cm square and were presented one at a time on the left half of the computer screen. Each picture was associated with a particular button on a Gamepad controller. A visual key to this pairing was displayed at all times on the right side of the computer screen, so that the pairings did not need to be memorized. As each picture appeared, the child had to press the button on the Gamepad controller that corresponded to the picture as quickly as possible. Although pictures in this task were presented one at a time, requiring the participant to make an additional cognitive step by matching the picture to the spatial location displayed on the on‐screen key, in all other ways the task was identical to the NV‐SRT.

##### Hebb serial order learning task (Hebb)

Following on seamlessly from the earlier immediate serial recall portion of the task, the implicit Hebb task introduced a covert repeated sequence in order to measure implicit learning of repeated sequences. There were 18 trials. Children were not told that the 6th, 9th, 12th, 15th and 18th trials were repetitions of the 3rd trial sequence. All 18 trials were the same sequence length, with the length of the sequence used for each child determined by their performance on the immediate serial recall task; the Hebb task sequence length was one item longer than the longest sequence the child had correctly recalled two or more times in the immediate serial recall task. The stimuli selected and their order of presentation were determined randomly. No stimulus appeared more than once in any sequence. Only items correctly recalled in the correct position were scored as correct (Conway et al., 2005). Points awarded per trial were, therefore, up to a maximum of the length of the list. Proportional scores for the blocks for the repeated and random sequences were calculated by dividing the raw score by the allocated list length. Higher proportional scores for repeated trials compared to random sequence trials were taken as evidence of implicit learning.

##### Contextual cueing task

**Figure 3:**
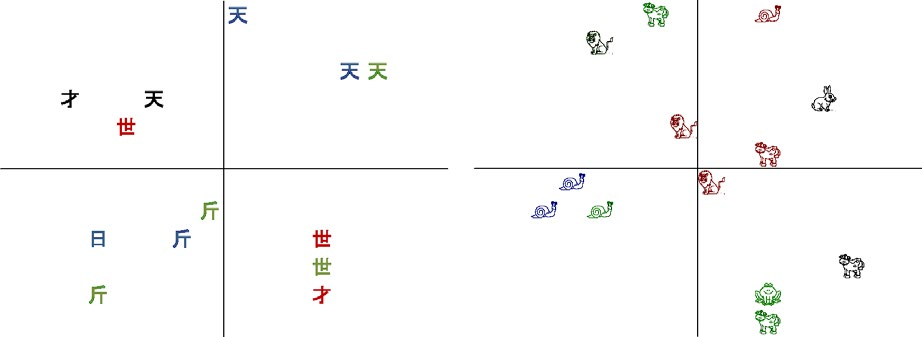
Example matrices for the non‐verbal and verbal conditions of the contextual cueing task

A dual condition contextual cueing task was used to measure visual search efficiency in both non‐verbal and verbal modalities simultaneously. Children were required to search for a target in matrices of distractor stimuli. They then had to indicate the quadrant of the matrix that the target appeared in as fast and accurately as possible, by pressing the key on the laptop keyboard that was associated with that quadrant, (A, Z, K or M; for a similar procedure, see Merrill et al., 2013). Five stimuli were chosen for each condition (verbal and non‐verbal): four distractor stimuli and one target stimulus. The verbal condition used line drawings of nameable pictures of familiar animals (frog, cow, rabbit, snail and lion). The non‐verbal condition required participants to discriminate between a simplified Chinese symbol and four other simplified Chinese symbols (see Figure 3). Both the symbols and the nameable pictures could appear in any of four colours (red, yellow, blue or green). All stimuli were 15 mm square.

All matrices displayed stimuli on invisible 12 × 12 grids divided into four easily identifiable quadrants. Three distractor stimuli appeared in each quadrant, such that 12 distractors and the target appeared in every matrix. For each participant the program randomly selected eight different locations to contain the target. Half of them were used in the verbal and half in the non‐verbal condition. These target locations were sampled from a set of five locations within each quadrant that were all approximately the same distance from the centre of the screen, such that one location was selected in each quadrant per condition. Distractors never appeared in the locations reserved for targets. Each target location was used for a different predictable matrix, resulting in four different predictable matrices for each condition. Target locations were selected in the same way for unpredictable matrices, but the arrangement of the distractors in each unpredictable matrix was always random and never repeated, so that the positions of distractors in these matrices could not aid visual search.

The experiment was divided into two phases. A learning phase of 80 trials included only predictable matrices, with each predictable matrix appearing once in each of 10 blocks. A testing phase of 128 trials subsequently compared speed of response on the ‘learned’ predictable matrices with an equal number of random unrepeated matrices where the position of the target was not predictable. There were eight blocks in the testing phase, with each block including the eight predictable matrices plus eight unpredictable matrices in random order. Each trial began with a 500‐ms fixation cross in the centre of the screen and children were instructed to focus on the cross each time it appeared. There was a 500‐ms ISI between trials. To keep accuracy throughout the task high, all errors were flagged. A single break was scheduled after 80 trials. The task took most children between 15 and 20 minutes to complete.

## 3 RESULTS

The means, standard deviations and reliabilities for all tasks are shown in Table 1.

**Table 1:** Performance on attainment and memory measures

	*N*	Mean	*SD*	Reliability
Age in months	101	98.31	3.84	–
Gender (f/m)	101	63/37	–	–
Handedness (right)	90	–	–	–
TROG‐2 (Blocks passed)	100	15.25	3.24	.88^s^
TROG‐2 (Total correct)	100	71.57	6.53	.88^s^
Literacy composite	101	.0006	.88	
WRAT‐3	100	12.11	2.84	.96^s^
PWM	100	37.76	10.89	
TOWRE‐2 Words	101	58.83	13.50	.90^r^
TOWRE‐2 Nonwords	101	33.93	12.67	.90^r^
Arithmetic composite	100	52.53	23.77	.97^r^
Addition	100	18.08	7.44	.92^r^
Addition plus carry	100	8.42	4.7	.89^r^
Subtraction	100	11.41	5.01	.88^r^
Subtraction plus carry	100	5.2	3.73	.85^r^
Multiplication	100	9.52	6.4	.93^r^
Dot comparison	100	13.14	5.53	.72^r^
Digit comparison	100	21.3	5.73	.80^r^
Dot count	100	11.03	2.78	.79^r^
WASI	100	17.93	5	.94^s^
Dot Locations (DL)				.76^s^/.57^r^
Learning	101	21.02	3.44	
Delay	100	5.29	1.17	
Consolidation	80	4.91	1.23	
Word Lists (WL)				.84^s^
Learning	98	32.81	5.64	
Delay	97	6.18	1.61	
Consolidation	76	5.72	1.73	
ISR (NV)	84	1.66	.397	.49^s^
ISR (V)	87	3.67	.78	.68^s^/.71^r^
NV‐SRT1 RT difference	98	58.57	48.49	.75^s^/.21^r^
NV‐SRT2 RT difference	90	89.4	48.47	.49^s^/.21^r^
V‐SRT1 RT difference	92	40.32	85.58	.17^s^/−.001^r^
V‐SRT2 RT difference	86	39.51	87.59	.27^s^/−.001^r^
Hebb NV	86	.062	.205	.5^s^
Hebb V	88	.088	.233	.58^s^/.29^r^
Contextual Cueing NV	100	.313	.415	−.03^s^
Contextual Cueing V	100	.248	.483	−.05^s^

^s^Split‐half reliability; ^r^test–retest reliability.

Attainment means were in line with test norms, where applicable. However, performance on the 15 words from the WRAT spelling test approached ceiling as did performance on the TROG‐2. Performance on the non‐verbal Dot Locations task was also high.

### 3.1 Learning on the implicit tasks

**Figure 4:**
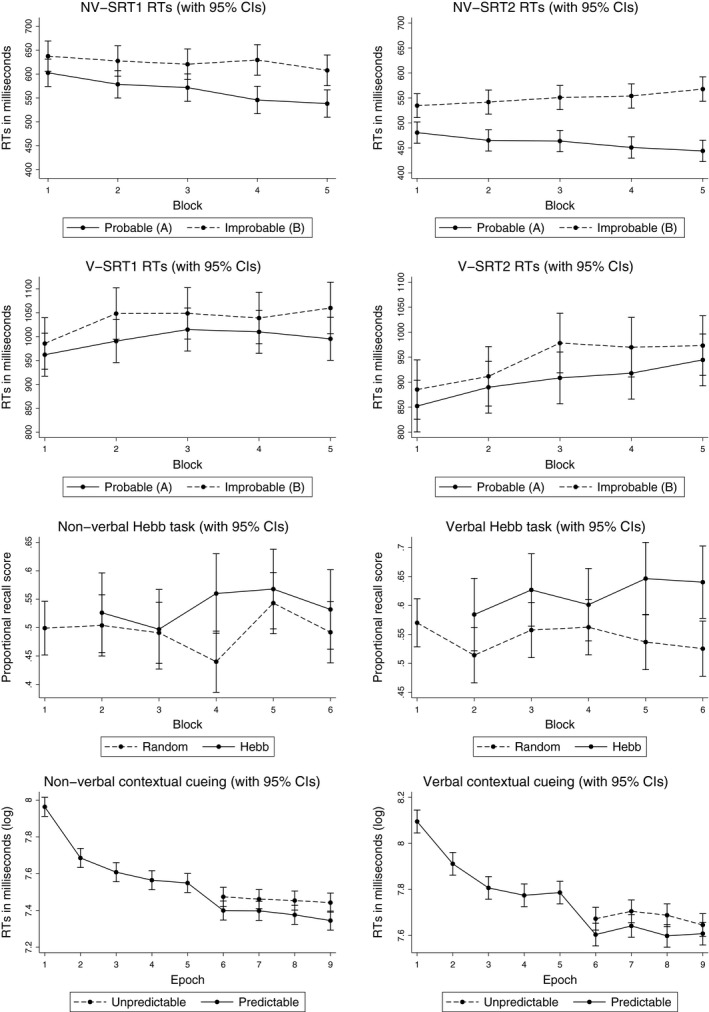
Response times for the SRT and contextual cueing implicit learning tasks, and recall scores for the Hebb tasks. Error bars are 95% confidence intervals

There was clear evidence of implicit learning on all tasks (see Figure 4). Mixed effects models (Rabe‐Hesketh & Skrondal, 2012) in Stata (13.0) were chosen to analyse response times (RTs) and recall scores for all implicit tasks in order to take account of item and participant variability. For all tasks sequence (or matrix) type, block (or epoch) and the interaction between them were entered as fixed effects and participants as a random effect. Reliability for the error statistics for the SRT and contextual cueing tasks was poor, so only RTs were analysed.

#### 3.1.1 SRT tasks

Inaccurate trials and trials over 5000 ms were removed and a moving criterion based on sample size (Selst & Jolicoeur, 1994) was used to remove remaining outlying observations. RTs for the improbable sequence were slower than for the probable sequence for all SRT attempts in every block. However, whereas RTs decreased over time on NV‐SRT, they increased over time on the verbal analogue, suggesting possible problems with attention and motivation on this task.

For both non‐verbal SRT attempts, RTs for the probable sequence were significantly faster than for the improbable sequence (NV‐SRT1: unstandardized regression coefficient = 34.766, *z *=* *4.41, *p *<* *.001, 95% CI [19.31, 30.22]; NV‐SRT2: unstandardized regression coefficient = 54.072, *z *=* *8.69, *p *<* *.001, 95% CI [41.88, 66.27]). On the first task attempt the interaction between block and sequence was significant for the last two blocks of the task, providing evidence of implicit learning (Block 4 unstandardized regression coefficient = 48.923, *z *=* *4.34, *p *<* *.001, 95% CI [26.84, 71.01]; Block 5 unstandardized regression coefficient = 34.751, *z *=* *3.06, *p *=* *.002, 95% CI [12.52, 56.98]). By the second attempt, this interaction was significant in every block (Block 2 unstandardized regression coefficient = 22.582, *z *=* *2.54, *p *=* *.011, 95% CI [5.15, 40.01]; Block 3 unstandardized regression coefficient = 33.026, *z *=* *3.69, *p *<* *.001, 95% CI [15.50, 50.55]; Block 4 unstandardized regression coefficient = 48.910, *z *=* *5.45, *p *<* *.001, 95% CI [31.32, 66.49]; Block 5 unstandardized regression coefficient = 69.673, *z *=* *7.61, *p *<* *.001, 95% CI [51.73, 87.61]). For the verbal SRT task, probable transitions were only significantly faster than improbable transitions on the second attempt (unstandardized regression coefficient = 33.02, *z *=* *20, *p *<* *.046, 95% CI [.61, 65.43]). The interaction between sequence and block failed to predict RT at any point in either verbal task.

#### 3.1.2 Hebb tasks

Mean recall for the repeating Hebb sequence was greater than for random sequences in both the non‐verbal and verbal versions on all blocks. The non‐verbal Hebb task did not show significant evidence of implicit learning, suggesting that the task demands with unnameable stimuli were too high. However, on the verbal task repeated Hebb sequences were recalled significantly better than random sequences (unstandardized regression coefficient = .115, *z *=* *3.21, *p *=* *.001, 95% CI [.045, .18]). The interactions were not significant.

#### 3.1.3 Contextual cueing task

Only RTs in the testing phase were analysed. All inaccurate responses, responses over 10,000 ms and RTs three test phase standard deviations above or below each participant's epoch mean were excluded from analysis. RTs were positively skewed, so analysis was conducted on log transformed RTs. Targets were identified significantly faster in predictable matrices than in random ones for both non‐verbal and verbal conditions (Non‐verbal: unstandardized regression coefficient = −.072, *z *= −3.69, *p *<* *.001, 95% CI [−.110, −.034]; Verbal: unstandardized regression coefficient = −.0067, *z *= −3.00, *p *=* *.003, 95% CI [−.11, −.023]). No other effects were significant.

### 3.2 Reliabilities

Reliabilities for all tasks are shown in Table 1. The scores for all declarative tasks were based on the number of items correct. Reliabilities for the declarative tasks were generally good.

Implicit learning tasks required the calculation of derived measures for each participant. For the SRT tasks we used the proportional mean difference in RT between sequence types across all trials. For the Hebb tasks a proportional difference taken across the last three blocks of the task was used. For the contextual cueing tasks a single overall facilitation measure was created for each condition (NV and V) that was the mean difference between predictable and unpredictable matrices across the entire testing phase. Unfortunately, as shown in Table 1, these derived measures had poor reliabilities. Details of the methods for calculating reliabilities and the rationale behind the selection of each task's difference score measure are described in Appendix A (see online Supporting Information).

### 3.3 Corr**e**lations

Correlations between all literacy measures were high (WRAT spelling, PWM reading test and TOWRE word and non‐word reading *r*s from .62 to .81). *Z*‐scores for these measures were summed to create a composite literacy measure. Correlations between all measures are shown in Table 2.

**Table 2:** Correlations between all attainment and memory measures

	1	2	3	4	5	6	7	8	9	10	11	12	13	14	15	16	17	18	19	20	21
1. Age (months)																					
2. TROG‐2	.14																				
3. Literacy composite	.26**	.49**																			
4. Arithmetic composite	.15	.38**	.50**																		
5. Non‐verbal IQ	.04	.36**	.25*	.12																	
6. DL Learning	.11	.36**	.18	.13	.34**																
7. DL Delay	.09	.32**	.13	.17	.34**	.77**															
8. DL Consolidation	.04	.37**	.21	.2	.38**	.68**	.62**														
9. WL Learning	.23*	.48**	.26**	.33**	.20	.29**	.22*	.25*													
10. WL Delay	.15	.30**	.24*	.25*	−.00	.10	.08	.16	.60**												
11. WL Consolidation	.22	.25*	.19	.24*	.14	.17	.17	.17	.59**	.79**											
12. ISR (NV)	−.18	.33*	.15	.14	.46**	.40**	.28**	.40**	.23*	.05	−.03										
13. ISR (V)	.18	.52*	.28*	.31**	.33**	.33**	.24*	.40**	.49**	.35**	.27*	.36**									
14. Contextual Cueing NV	.12	.11	.10	.20	−.02	−.07	−.11	−.02	.06	.02	.04	−.02	.02								
15. Contextual Cueing V	.09	−.02	.05	.11	−.01	.06	.08	−.02	−.04	.02	.22	.11	−.07	−.10							
16. NV‐SRT1	−.03	−.03	−.20*	−.06	−.05	−.07	−.09	−.18	.10	−.03	−.04	−.25*	.08	−.03	−.12						
17. NV‐SRT2	.03	.03	.16	.01	.13	.07	.10	−.01	.15	.19	.26*	−.01	−.01	−.18	.15	.21					
18. V‐SRT1	−.01	.01	.06	−.02	.15	−.09	−.05	−.17	.14	.28**	.38**	−.15	−.01	.06	.12	−.08	.24*				
19. V‐SRT2	.04	.01	−.04	−.03	.09	.06	.11	.10	−.10	−.12	−.09	.02	.07	−.10	−.12	.11	.20	−.00			
20. Hebb NV	.06	.05	−.01	.12	.03	.07	.03	−.04	.14	.04	.10	.10	−.04	.12	−.05	−.02	.03	−.12	−.14		
21. Hebb V	−.08	.13	.04	.10	.14	.07	.10	.14	.09	−.03	−.02	.23*	.14	−.08	.01	.09	.03	.14	−.00	−.15	

*p <* *.05; **p <* *.01.

Measures of literacy, language, counting and NVIQ showed moderate to strong correlations with each other as expected. Measures of declarative memory showed moderate correlations with literacy, and somewhat lower correlations with language (TROG‐2) and arithmetic. The declarative memory tasks correlated with each other broadly as expected, with the dot location memory measures correlating strongly with each other, as did the word list learning measures. Measures of immediate serial recall (both verbal and non‐verbal) showed moderate correlations with most of the other memory tasks, and with each other. Finally, the measures of procedural memory correlated weakly and non‐significantly with measures of attainment (language, literacy, and arithmetic) and poorly with each other, reflecting the poor reliability of these measures.

### 3.4 Effects of children's language background

It was important to check that the pattern of results obtained is not influenced by differences between monolingual children and those with English as an additional language (EAL). As shown in Table 3 there were no statistically significant differences in language attainment between the EAL and monolingual children after Bonferroni correction for multiple comparisons; and the EAL children actually performed slightly but non‐significantly better than the monolingual children on tests of word reading. Effect sizes for the TROG‐2 show that the level of grammatical proficiency demonstrated by the EAL children is lower than their English mother‐tongue counterparts. Twenty of the monolingual children scored over 75 out of 80 on the TROG‐2 task, compared to 11 of EAL children, who showed a greater range of scores. Crucially, correlations that included only the monolingual children showed the same pattern as those for the overall sample (see Appendix B in online Supporting Information for details).

**Table 3:** Language attainment means (*SD*s) by monolingual and EAL subgroups and *t*‐test comparisons

	Mean (*SD*)		*t*(df = 98)	*p*	*Cohen's d*
Attainment test	Monolingual (*n *=* *49)	EAL (*n *=* *52)			
TROG‐2 (Blocks passed)	15.85 (3.21)	14.69 (3.18)	1.81	.07	.36
Trog‐2 (Total correct)	73.02 (6.18)	70.23 (6.62)	2.17	.03	.43
WRAT‐3	12.12 (3.21)	12.10 (2.47)	.05	.96	.01
PWM	36.77 (11.73)	38.67 (10.07)	−.87	.38	−.17
TOWRE‐2 Words	57.65 (16.45)	60.04 (9.85)	−.81	.38	−.18

### 3.5 Confirmatory factor analysis

**Figure 5:**
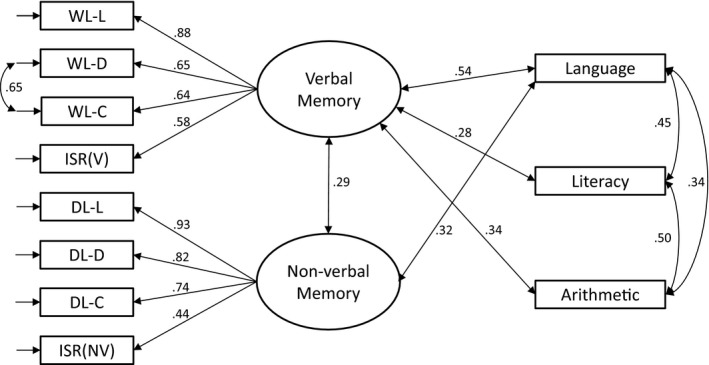
Confirmatory factor analysis showing relationship of memory and attainment tasks to latent variables of verbal and non‐verbal memory. WL‐L = Word Lists learning score; WL‐D = Word Lists delay score; WL‐C = Word Lists consolidation score; DL‐L = Dot Locations learning score; DL‐D* *= Dot Locations delay score; DL‐C* *= Dot Locations consolidation score: ISR(V) = verbal immediate serial recall; ISR(NV) = non‐verbal immediate serial recall; Language = TROG‐2 total score; Literacy = Literacy composite of WRAT spelling, TOWRE word and non‐word reading and Picture Word Matching; Arithmetic = composite of TOBANS addition, subtraction and multiplication subtests

Given the low reliabilities of the measures of implicit learning, and the low correlations between these measures, they were not considered further. A confirmatory factor analysis model for the eight declarative memory measures and measures of attainment was estimated in Mplus 7.4 (Muthén & Muthén, 1998–2016) with missing values being handled with Full Information Maximum Likelihood estimation. The model used is shown in Figure 5 and provides an excellent fit to the data (*χ* ^*2*^ (38) = 40.60, *p *=* *.356; RMSEA = .026 [90% CI .000–.076]; CFI = .99; TLI = .99). In this model the verbal and non‐verbal declarative memory measures define two separable factors which correlated moderately with each other (*r *=* *.29). The verbal factor correlated moderately with measures of attainment (language (TROG‐2) *r *=* *.54; literacy *r *=* *.28; arithmetic *r *=* *.34). The non‐verbal factor did not correlate significantly with literacy or arithmetic, but did correlate with language as measured by TROG‐2 (*r *=* *.32).

## 4 DISCUSSION

This study assessed claims that impairments in a procedural learning system are a causal risk factor for language learning deficits in children (dyslexia and language impairments; Nicholson & Fawcett, 2007; Ullman & Pierpont, 2005). In line with earlier findings, in our large sample of 7‐ to 8‐year‐old children, measures of verbal declarative memory showed adequate reliabilities and loaded on separable verbal and non‐verbal latent factors. Furthermore, variations in verbal declarative memory were stronger correlates of language, literacy and arithmetic skills than variations in non‐verbal declarative memory. In contrast, a range of widely used implicit learning tasks had poor reliabilities and showed no appreciable correlation with each other or with measures of attainment. Our results seriously question the suggestion that the construct of a ‘procedural learning system’ can be reliably measured and cast strong doubt on claims from earlier studies that deficits in such a system are related to language learning difficulties.

As documented in the introduction, many studies have reported deficits on a range of implicit learning measures in children with language impairment (Hedenius, 2013; Hsu & Bishop, 2014; Lum et al., 2010) or dyslexia (Howard et al., 2006; Vicari et al., 2005). However, the findings from previous studies are distinctly mixed, with many null results (Gabriel et al., 2011; Lum & Bleses, 2012; Majerus, 2009; Staels & Van den Broeck, 2015). Methodologically, most studies in this area share a number of undesirable characteristics: (1) the studies use extreme group designs; (2) sample sizes are small, giving low statistical power; (3) only a single measure, or a limited range of measures of learning and memory are used in any one study; (4) the studies do not report reliability estimates for the measures of learning.

Studies with low statistical power are likely to yield many false positive results (Button et al., 2013), and extreme group designs will tend to overestimate the extent of any true linear relationship between two variables in the population as a whole. Our solution to these problems was to administer a wide range of measures of procedural and declarative learning and language and attainment to a large and representative sample of children. We found clear evidence of learning in our procedural memory tasks – but such measures proved to have extremely low reliabilities, consistent with some previous evidence (Buchner & Wippich, 2000; Reber et al., 1991; Salthouse et al., 1999).

Why might the reliability of the procedural learning tasks be so low? Ostergaard (1998) noted that the relative contribution of learned information is likely to be far lower in procedural than declarative tasks. In a declarative task like word list recall, there is minimal external stimulus information for the participant to process at recall and hence variation in memory integrity is likely to cause most of the variance in performance. In a procedural task such as contextual cuing, in contrast, each trial evokes a number of perceptual as well as motoric processes that will contribute to variance in performance over and above learned sequence knowledge. If a target is embedded amongst 12 distractors in a contextual cuing display, for example, then variation in basic perceptual processes (scanning across the objects until the target is identified) and response selection and execution will all contribute to measured variance. Any relevant procedural information that can be retrieved from memory about the likely location of the target in a familiar display will make only a small contribution to the RT on a given trial. Ostergaard formalized this idea in his Information Availability model. When the relative contribution of learned information to performance is low, the reliability of the task for measuring that learned information will be low too.

One potentially important determinant of the reliability of any task is the number of trials used (Nunnally & Bernstein, 1994). The length of implicit learning tasks used in this study was similar to the length of tasks used by many others in the field. Serial reaction time tasks have occasionally used over 1000 trials (Rüsseler et al., 2006; Kelly et al., 2002), but they have often been much shorter, with some including as few as around 300 trials (Lum & Bleses, 2012; Menghini et al., 2006; Stoodley, Harrison, & Stein, 2006; Vicari et al., 2005). The length of contextual cueing tasks varies across studies, but evidence of cueing in children has been shown in tasks containing as few as 80 trials in total (Dixon et al., 2010). The number of Hebb repetitions used here was the same as in Hsu and Bishop (2014). The reliability of the implicit learning tasks in this study is, therefore, likely to be broadly comparable to the reliabilities of measures used in previous studies in this area. Future research should investigate whether increases in the number of trials used in procedural learning tasks such as those used here will result in estimates of learning with adequate reliability.

In addition, although children over the age of 6 years are able to cope with the demands of cognitive testing across multiple tasks, they are more prone to boredom and fatigue than adults (Luciana & Nelson, 2002), with resultant down‐stream effects on the quality of data they produce. For example, it has been demonstrated that children can be inconsistent performers on tasks such as Hebb learning compared to adults (Archibald & Joanisse, 2013; Mosse & Jarrold, 2008), which may explain the unreliable results on this task in particular.

Evidence from the current study seriously questions the viability of the procedural deficit hypothesis. It is clear, however, that in order to adequately test such a hypothesis more work will be required to develop measures of procedural learning with adequate reliabilities. If reliable measures can be developed, only then will we be in a position to adequately assess the procedural learning hypothesis. The mixed evidence to date for this hypothesis likely reflects the low statistical power (and unreliable measures) of studies in this area.

In contrast to our finding for procedural learning, our measures of declarative memory showed reasonable reliabilities and moderate correlations with measures of language skills and academic attainment. The correlation found here between our measure of verbal serial recall and measures of attainment are in line with many earlier findings. For example, Melby‐Lervåg, Lyster, and Hulme (2012) reported a robust correlation between measures of immediate verbal memory span and reading ability (pooled effect size estimate *r *=* *.34). Similarly, verbal free recall performance is typically poor in children with dyslexia or language impairment (Baird, Dworzynski, Slonims, & Simonoff, 2010; Kramer, Knee, & Delis, 2000). Such correlations may or may not reflect causal effects of declarative memory on the development of reading and language skills, since some have argued that phonological processing deficits and verbal memory impairments in dyslexia are two expressions of the same underlying problem (Tijms, 2004) and that verbal short‐term memory skills may be a by‐product of the mechanisms that subserve language itself (Hulme & Snowling, 2009; Acheson, Hamidi, Binder, & Postle, 2011; Allen & Hulme, 2006).

In summary, this study has shown that verbal declarative memory measures correlate with language attainment, yet in spite of considerable evidence of implicit learning on most implicit tasks, no relationship between implicit learning and language attainment was found. Crucially, the derived measures representing implicit learning displayed very low reliability. The development of implicit learning tasks with adequate reliability is needed, before any questions about the relationship between implicit procedural learning and language can be answered definitively.
