# Supplementary material for: The procedural learning deficit hypothesis of language learning disorders: we see some problems
Source: Dev Sci. 2017 Mar 2;21(2):e12552. doi: 10.1111/desc.12552 (PMC5888158; doi:10.1111/desc.12552)
Supplement: Supplementary file 1 [file DESC-21-na-s001.docx]

**Online Supporting Information**

Appendix A

**Calculating reliabilities for the implicit learning tasks**

As a result of the additive nature of measurement error, the difference between two cognitive test scores is less reliable than either of the scores it is derived from (Lord, 1958; Overall & Woodward 1975). It was, therefore, important in the current study to consider the merits of different scoring methods for implicit learning on a case-by-case basis.

**Serial reaction time tasks.** Both difference scores and regression-based residual measures were investigated. Difference scores were calculated by subtracting the task mean for improbable transitions from the task mean for probable transitions to give a measure that took account of the ratio between probable and improbable trials. Residual measures reflected the degree of deviation from the regression slope for probable transitions that occurred on improbable transitions. The difference score measure was preferred, as there was relatively little evidence of an interaction between the sequences on the majority of the SRT tasks. Split-half reliability was then calculated by sequentially numbering the trials for each sequence and calculating a proportional difference score measure for odd and evenly numbered trials separately. Test-retest reliability was established by correlating children’s difference scores on the first and second time of taking the task.

It should be noted that the residual measures demonstrated similar levels of unreliability and an equally nonsignificant relationship with language-related attainment. Additionally, an alternative, coarser-grained, binary measure of difference, first recommended by Lord (1958), that has been used for serial reaction time tasks with reported success in studies of individual differences in implicit learning was also considered (Kaufman et al., 2010; Pretz, Totz, & Kaufman, 2010). This method first calculated the effect size for the difference between the probable and improbable RT means for the sample (Cohen’s *d* for NV-SRT1 = .22; NV-SRT2 = .48; V-SRT1 = .08; V-SRT2 = .09) and allocated a point for each block a participant’s learning on the probable trials was as high or higher than this sample effect size. Test retest reliability was once more calculated, this time using the binary scores. Although reliability had improved, it was still unacceptably low (*r*’s for NV-SRT = .28; V-SRT *r* = .20). Nevertheless, relationships with both language-related and declarative measures were examined, but were low and none were significant once Bonferroni corrections had been made.

**Hebb serial learning tasks.** Recall scores were divided by list length, in order to control for variability in participant recall ability. Although the gradient of improvement on repeated trials compared to random trials has frequently been used to give an index of Hebb sequence learning (Guerard et al., 2011; Hitch, Flude, & Burgess, 2009; Hsu & Bishop, 2014; Page, Cumming, Norris, McNeil, & Hitch, 2013; Szmalec et al., 2011), this is an effective way of capturing implicit learning only if participants show stable recall on random trials and improved recall for the repeated sequence over time (Hebb, 1961). This is usually the case with adult participants, but children have been shown to exhibit a different pattern of Hebb learning to adults (Archibald & Joannise, 2008; Mosse & Jarrold, 2008), with inconsistent recall on random trials, combined with more consistent, rather than improved, recall of the repeated sequence. This pattern was evident in the current study and a more suitable difference score measure was, therefore, selected, which summed the difference in proportional recall across blocks 4 to 6, by which time any Hebb learning should have been established.

Split-half reliability could only be calculated for the random trials, correlating the first and second random trial per block, as the Hebb trials were not independent. To establish test-retest reliability a different sample of twenty-seven children (13 girls, 14 boys) were tested on the verbal ISR and Hebb learning task on two occasions 3 days apart. Mean age was 7 years and 7 months (*SD* = 3.97 months). As stimulus selection was randomised, children were presented with different Hebb, as well as random, sequences on each occasion. Mixed effects models with block and sequence as fixed effects and participant as a random effect showed evidence of significant implicit learning (Time 1: unstandardized regression coefficient = .143, *z* = 2.20, *p* = .028, 95% CI [.016, .271]; Time 2: unstandardized regression coefficient = .225, *z* = 3.48, *p* = .001, 95% CI [.098, .352]). Test-retest reliability was then calculated by correlating the proportional difference score across the last 3 blocks of the task at Time 1 and at Time 2.

**Contextual cueing task.** RT variability was controlled in the same way as in the SRT tasks. A difference score that captured the difference in RTs on predictable versus unpredictable matrices across the entire testing phase was calculated. Similar difference scores have been used in published research (Dixon et al., 2010; Brown, Aczel, Jiménez, Kaufman, & Grant, 2010). A measure that attempted to remove noise from the data by dividing each participant’s mean RT by their testing phase standard deviation, was also investigated, but did not improve reliability and the simpler difference score was, therefore, preferred. Split-half reliability was estimated by numbering trials sequentially by matrix type and calculating difference scores for odd and evenly numbered trials separately. These difference scores were then correlated to give an index of split-half reliability. This was done for verbal and non-verbal conditions of the task separately.

Appendix B

**English as an Additional Language (EAL) matching**

We examined the relationships between outcome measures and predictors and found no evidence of meaningful group differences between the EAL fluent English speakers and monolingual (EMT) children, other than the monolingual group exhibited even stronger relationships between verbal declarative memory and the language (TROG-2) measure than the sample as a whole (verbal free recall score (monolingual *r* = .52; overall *r* = .48); verbal ISR learning score (monolingual *r* = .71; overall *r* = .52)), while the non-verbal declarative free recall scores ceased to be significant (see table S1). The implicit learning measures for the monolingual group still failed to correlate significantly with language or with each other. This is consistent with the study’s findings that it is specifically verbal declarative memory that relates to language, demonstrating that the pattern of results in the main study is not an artefact of having EAL fluent English speakers in the sample. It should be noted that the relationship between language and non-verbal IQ was also stronger for monolingual children than for the overall sample (monolingual *r* = .53; overall *r* = .36), highlighting a possible moderating influence of non-verbal IQ on both language and short-term verbal declarative memory.

*Table S1*

Correlations between language (TROG-2) and other measures by subgroup and overall sample.

| Measures | TROG-2 | | |
| --- | --- | --- | --- |
|  | Complete Sample  (n = 101) | EMT  (n = 49) | EAL  (n = 52) |
| Literacy composite | .49** | .58** | .46 |
| Arithmetic composite | .38** | .38 | .44 |
| WASI | .36** | .53* | .26 |
| Dot Locations (DL) |  |  |  |
| Learning | .36** | .33 | .44 |
| Delay | .32** | .23 | .39 |
| Consolidation | .37** | .41 | .39 |
| Word Lists (WL) |  |  |  |
| Learning | .48** | .52* | .51* |
| Delay | .30** | .39 | .24 |
| Consolidation | .25* | .41 | .13 |
| ISR (NV) | .33* | .41 | .31 |
| ISR (V) | .52* | .71** | .36 |
| NV-SRT1 RT difference | -.03 | -.29 | .14 |
| NV-SRT2 RT difference | .03 | -.05 | .12 |
| V-SRT1 RT difference | .01 | .04 | -.06 |
| V-SRT2 RT difference | .01 | -.11 | -05 |
| Hebb NV | .05 | -.12 | .20 |
| Hebb V | .13 | -.13 | .28 |
| Contextual Cueing NV | .11 | .21 | .06 |
| Contextual Cueing V | -.02 | .12 | -.07 |
| *p < .05; **p < .01; Bonferroni corrections applied | | | |

**References (for Online Supporting Information)**

Archibald, L. M., & Joanisse, M. F. (2013). Domain-specific and domain-general constraints on word and sequence learning. *Memory & Cognition*, *41*(2), 268-280.

Brown, J., Aczel, B., Jiménez, L., Kaufman, S. B., & Grant, K. P. (2010). Intact implicit learning in autism spectrum conditions. *Quarterly Journal of Experimental Psychology*, *63*(9), 1789-1812.

Hitch, G. J., Flude, B., & Burgess, N. (2009). Slave to the rhythm: Experimental tests of a model for verbal short-term memory and long-term sequence learning. *Journal of Memory and Language*, *61*(1), 97-111.

Kaufman, S. B., DeYoung, C. G., Gray, J. R., Jiménez, L., Brown, J., & Mackintosh, N. (2010). Implicit learning as an ability. *Cognition*, *116*(3), 321-340.

Lord, F. M. (1958). The utilization of unreliable difference scores. *Journal of Educational Psychology*, *49*(3), 150.

Mosse, E. K., & Jarrold, C. (2008). Hebb learning, verbal short-term memory, and the acquisition of phonological forms in children. *The Quarterly Journal of Experimental Psychology*, *61*(4), 505-514.

Overall, J. E., & Woodward, J. A. (1975). Unreliability of difference scores: A paradox for measurement of change. *Psychological Bulletin*, *82*(1), 85.

Page, M. P., Cumming, N., Norris, D., McNeil, A. M., & Hitch, G. J. (2013). Repetition-spacing and item-overlap effects in the Hebb repetition task. *Journal of Memory and Language*, *69*(4), 506-526.

Pretz, J. E., Totz, K. S., & Kaufman, S. B. (2010). The effects of mood, cognitive style, and cognitive ability on implicit learning. *Learning and Individual Differences*, *20*(3), 215-219.
